# Calcium-mediated regulation of mitophagy: implications in neurodegenerative diseases

**DOI:** 10.1038/s44324-025-00049-2

**Published:** 2025-02-03

**Authors:** Fivos Borbolis, Christina Ploumi, Konstantinos Palikaras

**Affiliations:** Department of Physiology, Medical School, National and Kapodistrian University of Athens, Athens, Greece

**Keywords:** Mitochondria, Metabolic disorders

## Abstract

Calcium signaling plays a pivotal role in diverse cellular processes through precise spatiotemporal regulation and interaction with effector proteins across distinct subcellular compartments. Mitochondria, in particular, act as central hubs for calcium buffering, orchestrating energy production, redox balance and apoptotic signaling, among others. While controlled mitochondrial calcium uptake supports ATP synthesis and metabolic regulation, excessive accumulation can trigger oxidative stress, mitochondrial membrane permeabilization, and cell death. Emerging findings underscore the intricate interplay between calcium homeostasis and mitophagy, a selective type of autophagy for mitochondria elimination. Although the literature is still emerging, this review delves into the bidirectional relationship between calcium signaling and mitophagy pathways, providing compelling mechanistic insights. Furthermore, we discuss how disruptions in calcium homeostasis impair mitophagy, contributing to mitochondrial dysfunction and the pathogenesis of common neurodegenerative diseases.

## Introduction

Calcium is one of the major signaling molecules, capable of modulating a multitude of cellular processes that span from neurotransmitter release and muscle contraction to gene expression and cell fate determination. The multifaceted role of calcium ions (Ca^2+^) in controlling such diverse cellular functions is achieved through the precise spatiotemporal and dynamic coordination of numerous effector proteins within distinct subcellular compartments. Each cell type expresses a unique repertoire of components, including Ca^2+^-binding proteins, pumps and channels, to establish specialized signaling systems that are dynamically fine-tuned to meet specific physiological demands across various organelles^1–4^. Among these, mitochondria stand out as critical regulators of calcium homeostasis^5^. Apart from their main role as energy suppliers, mitochondria serve as crucial hubs for Ca^2+^ signaling. Ca^2+^ buffering in mitochondria is pivotal for the maintenance of mitochondrial integrity, energy production, redox balance and apoptosis^6^. While moderate Ca^2+^ influx into mitochondria supports ATP synthesis and metabolic regulation, excessive mitochondrial Ca^2+^ accumulation can lead to oxidative stress, mitochondrial membrane permeabilization and the release of pro-apoptotic factors, ultimately leading to cellular damage^7–9^. Therefore, a balanced interplay between Ca^2+^ and mitochondrial quality control mechanisms is crucial for preserving cellular health and viability.

The role of Ca^2+^ in the regulation of mitophagy, the selective autophagic removal of damaged or superfluous mitochondria, has recently gained significant attention, particularly in the context of neurodegenerative diseases^10^. Dysregulated Ca^2+^ signaling can disrupt the process of mitophagy, resulting in the accumulation of defective mitochondria. This mitochondrial buildup exacerbates oxidative stress and promotes the activation of cell death pathways, culminating in cellular dysfunction and death. Such disturbances in calcium-regulated mitochondrial quality control mechanisms are increasingly recognized as major contributors to neurodegeneration^11–13^.

This review aims to provide a comprehensive overview of the intricate relationship between Ca^2+^ signaling and mitophagy pathways. By discussing the molecular mechanisms linking Ca^2+^ to mitochondrial quality control, we aim to explore how disruptions in these processes contribute to the pathogenesis of common neurodegenerative disorders, including Alzheimer’s, Parkinson’s and Huntington’s diseases, among others.

## General mitophagy mechanisms

Mitophagy is a finely tuned process that can be initiated either physiologically upon developmental or metabolic cues to remove superfluous mitochondria or conditionally as a mitochondrial quality control mechanism to eliminate dysfunctional organelles. The recognition of mitochondria targeted for degradation is mediated by specialized receptor and adaptor molecules, which orchestrate the recruitment of the autophagic machinery through ubiquitin-dependent or ubiquitin-independent mechanisms^14–17^.

### Ubiquitin-dependent mitophagy mechanisms

The PINK1/Parkin pathway, involving the kinase PTEN Induced Kinase 1 (PINK1) and the E3 ubiquitin ligase Parkin, is one of the well-studied ubiquitin-dependent mechanisms for mitophagy. Mutations in PINK1 and Parkin genes account for more than 50% of the autosomal recessive juvenile parkinsonism cases, while their abnormal expression has also been associated with other common neurodegenerative diseases^18,19^. In healthy mitochondria, with relatively high membrane potential (Δψ_m_), PINK1 is transported into the inner mitochondrial membrane (IMM) by the general mitochondrial import machinery, composed of the translocase of the outer membrane (TOM)/translocase of the inner membrane (TIM) complexes^20^. Once in the IMM, PINK1 undergoes proteolytic cleavage by mitochondrial processing peptidase (MPP), presenilin-associated rhomboid-like (PARL) and AFG3-like AAA ATPase 2 (AFG3L2) proteases, producing a 52 kDa PINK1 fragment that is released to the cytosol and rapidly targeted for degradation through the ubiquitin proteasome system (UPS)^20–23^. Interestingly, the truncated PINK1 cytosolic fragments, apart from being degraded, are suggested to interact with cytosol-resided Parkin, thus blocking its mitochondrial translocation and mitophagy initiation^24^.

In mitochondria with reduced Δψ_m_, the translocation of PINK1 to the IMM and its subsequent processing are blocked. In turn, full-length PINK1 is stabilized and forms homodimers at the outer mitochondrial membrane (OMM)^25^. PINK1 stabilization can also be triggered by unfolded protein stress in mitochondrial matrix, initiating mitophagy even in energetically healthy mitochondria^26^. Interestingly, recent studies revealed a neuron-specific mechanism for the PINK1/Parkin pathway. Specifically, PINK1 mRNA is co-transported with mitochondria in axons and dendrites, enabling localized PINK1 translation and ensuring the efficient degradation of damaged mitochondria in distal neuronal compartments^27,28^. Autophagy And Beclin 1 Regulator 1 (AMBRA1), initially identified as positive autophagy regulator, plays a key role in the stabilization of PINK1. Particularly, upon mitochondria depolarization, AMBRA1 associates with PINK1 at the OMM and prevents its mitochondrial import and subsequent degradation by interacting with TOM and ATPase Family AAA Domain Containing 3A (ATAD3A)^29^.

Upon autophosphorylation, PINK1 phosphorylates ubiquitin molecules and cytosolic Parkin, facilitating the recruitment of the latter to the OMM and activating its E3 ubiquitin ligase activity^30–39^. Although Parkin translocation has been extensively studied in non-neuronal cells, it also occurs in neurons, albeit at a slower and more compartmentalized manner, particularly in somatodendritic regions^40^. Parkin activation triggers a feed-forward loop, whereby it catalyzes the formation of ubiquitin chains on several OMM-localized proteins. These ubiquitin chains are then phosphorylated by PINK1, further enhancing the retention of additional Parkin molecules at the OMM and the expansion of ubiquitin tags for mitochondrial degradation^33,37^. Importantly, the nature of ubiquitin chains determines the fate of targeted mitochondrial proteins. While K48-linked chains typically signal for proteasomal degradation, K63-linked chains recruit autophagy adaptors, thereby facilitating mitophagy. In addition to K48- and K63, Parkin also assembles K6 and K11-linked ubiquitin chains, the role of which in the mitophagy process still remains elusive^41,42^. Among the numerous OMM proteins that serve as Parkin substrates are mitofusins 1 and 2 (MFNs)^43,44^, voltage-dependent anion channel 1 (VDAC1)^45,46^ and the mitochondrial Rho GTPase 1 (Miro1), also known as RHOT1^47^. Ubiquitination of the particular OMM-resident proteins not only marks the mitochondrion for degradation but also enhances the removal of fusion- and motility-related proteins, thereby isolating damaged mitochondria from the healthy mitochondrial population.

While Parkin is a major contributor to the ubiquitination of OMM proteins, several other E3 ligases are reported to function redundantly, including glycoprotein 78 (Gp78)^48^, mitochondrial ubiquitin ligase 1 (MUL1)^49^, Ariadne RBR E3 Ub protein ligase 1 (ARIH1)^50^, synphilin-1 recruited seven in absentia homolog 1 (SIAH-1)^51^, and HECT, UBA And WWE Domain-Containing Protein 1 (HUWE1)^52^. Among these, HUWE1 requires AMBRA1 as a cofactor and ubiquitinates MFN2, preventing mitochondrial fusion prior to mitophagy. Notably, AMBRA1 has a functional LC3-interacting region (LIR) motif that facilitates selective sequestration of ubiquitinated mitochondria into autophagosomes^53^. Given AMBRA1’s role in PINK1 stabilization^29^, AMBRA1 stands as a crucial ubiquitin-dependent mitophagy inducer irrespectively of the presence or absence of Parkin.

The next critical step for mitophagy involves the recognition of the ubiquitin-tagged OMM proteins by specialized adaptors, which engage the marked mitochondria with the autophagic machinery. These adaptor proteins, including optineurin (OPTN), neighbor of BRCA 1 (NBR1), Nuclear Dot Protein 52 (NDP52) and sequestosome (SQSTM1)/p62, possess ubiquitin-binding domains and LIR motifs for direct interaction with the lipidated form of microtubule-associated protein 1A/1B-light chain 3 (LC3) in the autophagosomal membranes^46,54–57^. Numerous studies in human cell lines have pointed out the crucial role of TANK-binding kinase 1 (TBK1) in the regulation of mitophagy. Following PINK1/Parkin-dependent ubiquitination in response to mitochondrial damage, TBK1 is activated and phosphorylates several adaptor proteins, including OPTN, NDP52 and SQSTM1, enhancing their affinity for ubiquitinated substrates and promoting their association with autophagosomes^58^. Beyond the phosphorylation of autophagy adaptors, TBK1 has additional roles for mitophagy enhancement. TBK1-dependent phosphorylation of RAB7 at Ser72 facilitates ATG9 recruitment, contributing to the de novo synthesis of autophagosomal membranes^59^. Additionally, RAB7 phosphorylation disrupts its interaction with Rubicon, a negative regulator of autophagy, while promoting its association with Pacer, a positive regulator for Parkin-dependent mitophagy^60^. Beyond RAB7, TBK1 also phosphorylates LC3 and GABA Type A Receptor-Associated Protein (GABARAP) proteins preventing their premature cleavage by ATG4^61^. Notably, a very recent study revealed that TBK1 undergoes K63-linked polyubiquitination by the E3 ligase Tripartite Motif-Containing Protein 5 Alpha (TRIM5α). This modification promotes the assembly of a self-amplifying platform, composed of TRIM5α, ΤΒΚ1 and mitophagy adaptors, facilitating the process of mitophagy^62^. Additionally, another study demonstrated that TBK1 can be K63-polyubiquitinated by Parkin in ischemic hippocampal CA1 neurons following hypoxic post-conditioning, leading to significantly enhanced mitophagy. Further studies are required to investigate the physiological role of TBK1, if any, in neuronal mitophagy.

### Ubiquitin-independent mitophagy mechanisms

In addition to ubiquitin-mediated mitophagy, ubiquitin-independent mechanisms for the selective degradation of mitochondria have also been characterized. These mechanisms rely on mitochondria-localized receptors of protein or lipid nature, which, upon specific stimuli, interact directly with autophagosomes bypassing the need for ubiquitination.

The pro-apoptotic proteins BCL2 and adenovirus E1B 19-kDa-interacting protein 3 (BNIP3) and BNIP3-like (BNIP3L) [also known as NIP3-like protein X (NIX)] have been identified as functional mitophagy receptors^63–66^. The expression of both proteins is highly induced upon hypoxic conditions in a hypoxia-inducible factor (HIF)-dependent manner^67,68^. Beyond its role in hypoxia, BNIP3L/Nix is vital for programmed mitophagy events during development. Specifically, it is required for the elimination of mitochondria during the differentiation of erythrocytes, retinal ganglion cells (RGCs), macrophages, keratinocytes, cardiac and oligodendrocyte progenitor cells, thereby supporting cellular maturation and function in these distinct contexts^69–73^. Moreover, BNIP3-mediated mitophagy is essential for maintaining pluripotency in embryonic stem cells (ESCs)^74^. Since BNIP3 and BNIP3L/NIX are ubiquitously expressed and distributed evenly at the OMM, numerous studies have explored their activation mechanisms to stimulate mitophagy. Phosphorylation at key residues has been shown to enhance their binding to LC3 and GABARAP proteins, facilitating mitophagosome formation. UNC51-like kinase-1 (ULK1), the catalytic kinase of the autophagy pre-initiation complex, has recently been reported to phosphorylate serine residues adjacent to the N-terminal LIR domains of BNIP3 and BNIP3L/NIX, thereby inhibiting their proteasomal degradation and enhancing their interaction with LC3^75,76^. Notably, BNIP3L/NIX phosphorylation in its C-terminal transmembrane domain impedes its ability to form homodimers in the OMM, negatively impacting LC3 binding and mitophagy progression^77^. Furthermore, the OMM-localized Phosphatase Targeting COQ7 (PPTC7) has recently emerged as a negative regulator of BNIP3 and BNIP3L/NIX, enhancing their proteasomal degradation and thereby restraining basal mitophagy^78^. Several studies have provided evidence for the interconnection of BNIP3- and NIX-mediated mitophagy with the PINK1/Parkin pathway. BNIP3 interaction with PINK1 has been shown to stabilize the retention of the latter to the OMM by suppressing its proteolytic cleavage^79^. Additionally, BNIP3L/NIX is required for Parkin translocation and mitophagy execution upon carbonyl cyanide m-chlorophenyl hydrazone (CCCP)-induced mitochondria depolarization^80^. Notably, studies in mammalian cells, *Drosophila melanogaster* and the nematode *Caenorhabditis elegans* have shown that BNIP3L/NIX is itself a substrate of Parkin, and its ubiquitination promotes the recruitment of autophagy adaptors, enhancing PINK1/Parkin-mediated mitophagy^81,82^. Interestingly, a recent study revealed that BNIP3 and BNIP3L/NIX are constitutively delivered to lysosomes through an autophagy-independent mechanism, involving the endolysosomal and UPS systems, highlighting additional layers of control in BNIP3-associated mitophagy and its impact on cellular physiology^83^.

FUN14 domain-containing protein 1 (FUNDC1) is an integral OMM protein that functions as a mitophagy receptor, particularly under hypoxia^84,85^. Several regulatory mechanisms have been reported to modulate FUNDC1-dependent mitophagy. Under non-stress conditions, Src and casein kinase-2 kinases phosphorylate FUNDC1 at Tyr18 and Ser13 respectively, impeding its mitophagy-inducing activity. In response to hypoxia or mitochondrial uncoupling, Phosphoglycerate Mutase Family Member 5 (PGAM5), a mitochondrially-localized phosphatase mediates the dephosphorylation of FUNDC1 at Ser13, thereby triggering mitophagy. This process is negatively regulated by the anti-apoptotic BH3-protein Bcl2 like 1 (BCL2L1), which inhibits PGAM5, thus preventing FUNDC1 dephosphorylation and suppressing mitophagy^86^. In response to hypoxia, phosphorylation of FUNDC1 by ULK1 kinase enhances significantly its interaction with LC3^87^. Beyond its direct role in mitophagy, FUNDC1 has been implicated in the regulation of mitochondrial dynamics by interacting with key regulatory proteins, promoting mitochondrial fragmentation prior to mitophagy^88^.

The pro-apoptotic Bcl-2-like protein 13 (BCL2L13), the mammalian ortholog of yeast Atg32, has been identified as a mitophagy receptor, possessing a functional LIR motif^89^. Mechanistically, upon mitochondria depolarization, BCL2L13 binds to and attracts LC3B to the OMM, facilitating the recruitment of the ULK1 complex and the subsequent interaction of the latter with LC3B^90^. Similarly to FUNDC1 and BNIP3 receptors, the LC3-interacting activity of BCL2L13 is controlled by phosphorylation events^89^. However, the kinases and/or phosphatases responsible for modulating its mitophagic activity have not been identified yet. FK506‐binding protein 8 (FKBP8) is an OMM anti-apoptotic protein that also functions as a mitophagy receptor via its N-terminal LIR motif^91^. Remarkably, during Parkin-mediated mitophagy FKBP8 translocates from mitochondria to the ER, preventing its degradation and supporting cell survival^92^. This selective relocation also occurs, albeit to a lesser extent, during Parkin-independent mitophagy^91^.

In addition, several mitophagy receptors that are not embedded in the OMM have also been identified. Spermatogenesis Associated 33 (SPATA33) functions as a mitophagy mediator by directly binding to the LC3-conjugation component ATG16L1 and the OMM channel VDAC2. This dual interaction allows SPATA33 to selectively eliminate mitochondria in male germline cells^93^. A very recent study revealed that mitochondrial protein 18 (MTP18), an integral inner mitochondrial membrane protein, possesses a functional LIR motif, essential for inducing mitophagy and supporting survival in oral cancer cell lines. Its ability to function as a mitophagy receptor highly depends on mitochondrial fission and Parkin-mediated rupture of the OMM which exposes MTP18’s LIR domain and facilitates its interaction with LC3^94^. In a similar manner, upon Parkin-dependent rupture of the OMM, the LIR motif of the IMM-resided protein prohibitin 2 (PHB2) is exposed, enabling its interaction with LC3. PHB2 is required for Parkin-induced mitophagy in mammalian cells and for the elimination of paternal mitochondria following embryonic fertilization in *C. elegans*^95^. ATAD3B is another IMM protein recently identified to function as a mitophagy receptor. Under normal conditions, ATAD3B forms hetero-oligomers with ATAD3A within the IMM, contributing to mitochondrial DNA (mtDNA) stability. However, in response to oxidative stress or mtDNA damage, ATAD3A-ATAD3B interaction is disrupted, leading to the retention of ATAD3B at the OMM. In this altered localization, the unique C-terminal LIR motif of ATAD3B becomes exposed, facilitating its interaction with LC3B and promoting the mitophagic clearance of damaged mitochondria^96^.

Emerging evidence suggests that certain lipids can bind directly to LC3-decorated autophagosomes and serve as mitophagy receptors. Based on research conducted in cell lines and primary cortical neurons, in response to mitochondrial damage or depolarization, cardiolipin (CL), a dimeric phospholipid predominantly localized at the IMM, is actively externalized to the OMM, where it directly associates with lipidated LC3 to initiate mitophagy. CL translocation is catalyzed by the enzymatic activity of nucleoside diphosphate kinase-D (NDPK-D), mitochondrial creatine kinase (MtCK) and phospholipid scramblase 3 (PLS3)^97,98^. While the exact mechanisms activating these enzymes remain unclear, PLS3 phosphorylation by protein kinase C-δ (PKC-δ) may also be critical for CL translocation^99^. Beyond its well-characterized role as a structural component of cellular membranes, the sphingolipid C(18)-ceramide has been also recognized as a mitophagy receptor that promotes autophagic cell death in human cancer cells^100^. Upon stress-induced nitrosylation and activation of the fission protein Dynamin-Related Protein 1 (DRP1), ceramide synthase 1 (CerS1) is translocated from the endoplasmic reticulum (ER) to the OMM, where it synthesizes C(18)-ceramide, which, in turn, binds directly to LC3B-II, inducing mitophagy and cell death^101^.

## Calcium homeostasis and intracellular trafficking

**Fig. 1:**
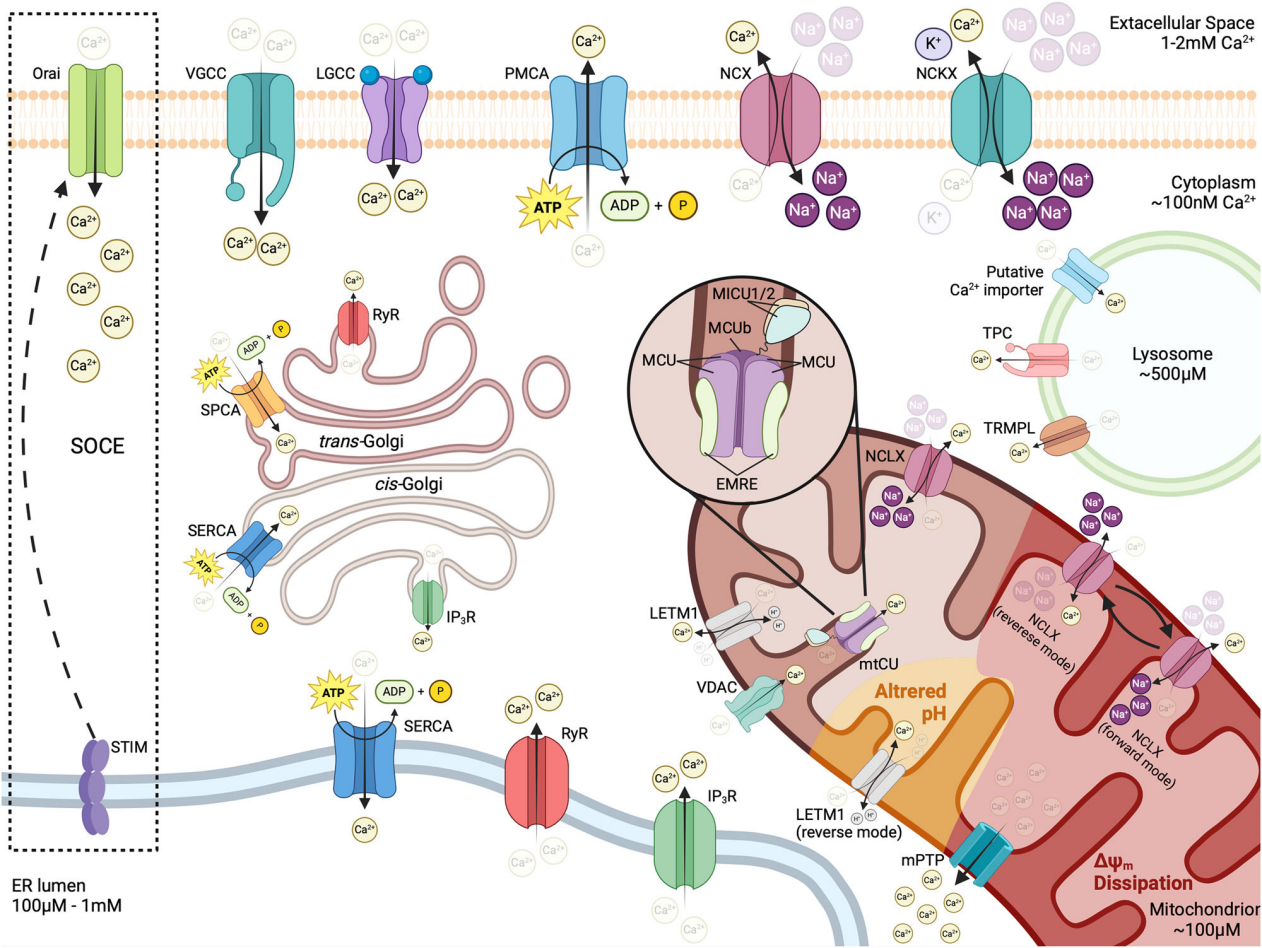
Intracellular Ca^2+^ trafficking. Ca^2+^ ions are constantly transferred from the cytoplasm to the extracellular space, through the action of the plasma membrane Ca^2+^ ATPase PMCA and Na^+^/Ca^2+^ (NCX) or Na^+^/Ca^2+^-K^+^ (NCKXs) exchangers. Opening of voltage-gated or ligand-gated plasma membrane Ca^2+^ channels (VGCCs or LGCCs) results in the rapid influx of Ca^2+^ ions along their chemical gradient. Inside the cell, large amounts of Ca^2+^ are transferred in the ER by the sarcoendoplasmic reticulum Ca^2+^-ATPase (SERCA) and can be released upon stimulation via IP_3_R or RyR channels. Depletion of ER Ca^2+^ stores induces the translocation of STIM protein to ER/PM contact sites where it activates the Ca^2+^ channel Orai to mediate prolonged influx of extracellular Ca^2+^. Ca^2+^ is also stored in the *cis*-Golgi through its transfer by SERCA or the *trans-*Golgi via the Ca^2+^-ATPase SPCA and can be released through IP_3_R or RyR channels, respectively. The Ca^2+^ content of lysosomes, built up by the transfer of ions from the cytoplasm via an unidentified importer, can be released through the action of transient receptor potential mucolipin channels (TRPML) and two-pore channels (TPC). The OMM is easily penetrated by Ca^2+^ ions that pass through VDAC but has to be transferred by the highly selective mitochondrial Ca^2+^ uniporter (mtCU) complex of the IMM in order to reach the matrix. Ca^2+^ export from mitochondria is mediated by the Na^+^/Ca^2+^ exchanger NCLX and the Ca^2+^/H^+^ antiporter Leucine Zipper And EF-Hand Containing Transmembrane Protein 1 (LETM1). Ca^2+^ overload or dissipation of Δψ_m_ can induce the opening of the mitochondrial permeability transition pore (mPTP), which mediates the rapid efflux of Ca^2+^ to the cytoplasm. Depolarization (shown in red) or altered pH (shown in yellow) can allow the reversal of NCLX or LETM1, respectively, transforming them into Ca^2+^ importers that function with different properties than the mtCU. For details see text. Created in BioRender. Palikaras, K. (2024) https://BioRender.com/v71t847.

Calcium ions (Ca^2+^) have a unique role in eukaryotic systems, as they affect multiple signaling pathways, including the phospholipase C (PLC), the protein kinase C (PKC) and the calmodulin/calcineurin pathways, to modulate vital cellular processes, such as gene expression, differentiation, metabolism and cell death^102^. At the same time, Ca^2+^ also mediates the establishment of membrane potentials and the induction of electrical signals that control the function of excitable cells, like neurons and muscles. To sustain this functional versatility, cells employ a variety of calcium channels that serve to tightly control Ca^2+^ levels in the cytoplasm and spatially constricted cytoplasmic subregions, or within organelles that also function as Ca^2+^ stores, mainly the endoplasmic/sarcoplasmic reticulum (ER/SR), the Golgi apparatus (GA), mitochondria and lysosomes (Fig. 1). Below we present the most important routes of intracellular Ca^2+^ trafficking that function to maintain its homeostasis and/or elicit the appropriate responses to changing conditions.

### Cytoplasmic calcium

Under resting conditions, cytoplasmic calcium concentrations ([Ca^2+^]_c_) are kept at low levels, typically around 10^−^^7^ M (100 nM), which is 10,000–20,000 times lower than the approximate extracellular [Ca^2+^] of 1–2 mM. Such a low [Ca^2+^]_c_ is maintained by the action of the plasma membrane (PM) Ca^2+^ ATPase (PMCA), which consumes the energy of ATP hydrolysis to pump Ca^2+^ outside of the cell against its gradient, and the function of Na^+^/Ca^2+^ exchanger (NCX) and Na^+^/Ca^2+^-K^+^ exchangers (NCKXs), that export 1 Ca^2+^ in exchange for the entry of 3 Na^+^, or 1 Ca^2+^ and 1 K^+^ in exchange for 4 Na^+^ ions respectively, using the relatively low cytoplasmic [Na^+^] as a driving force^103^. The establishment of low [Ca^2+^]_c_ is further facilitated by the activity of the sarcoendoplasmic reticulum Ca^2+^-ATPase (SERCA), which pumps Ca^2+^ inside the ER/SR and the GA and, to a lesser extent, by the mitochondrial Ca^2+^ uniporter (mtCU) that mediates calcium uptake by mitochondria^103^. A similar supporting role has also been suggested for lysosomal Ca^2+^ uptake, however the exact molecular players that mediate calcium import inside these organelles are only just starting to emerge^104^.

The low resting [Ca^2+^]_c_ that is established through the aforementioned mechanisms, provides a massive driving force for calcium entry inside the cell, which is further enhanced by the electrical potential that exists across the PM (Δψ = −70 mV in the cytoplasm side relative to the extracellular milieu) and is opposed only by the latter’s impermeability. As a result, the opening of PM Ca^2+^ channels leads to the rapid influx of calcium inside the cell, following its electrochemical gradient, and causes a dramatic increase of [Ca^2+^]_c_, that can go beyond the 1 μM range. Although the identity of such channels varies among cell types, they are generally categorized as either voltage-gated Ca^2+^-permeable channels (VGCCs), activated by membrane depolarization, or ligand-gated Ca^2+^-permeable channels, activated by the binding of specific signaling molecules^103,105^. Likewise, cytoplasmic calcium levels can rise through the release of Ca^2+^ from internal stores, a feature that allows the generation of spatially confined calcium spikes in discrete cellular regions through localized discharges.

### Calcium fluxes in the ER/SR and Golgi apparatus

The ER/SR is the predominant source of internal Ca^2+^, with a luminal concentration that typically ranges between 100 μM and 1 mM, that is 1000–10,000 times higher than the average [Ca^2+^]_c_. ER/SR releases Ca^2+^ mainly via the inositol-1,4,5-triphosphate receptors (IP_3_R), activated by PM G protein-coupled receptor (GPCR) signaling, and ryanodine receptors (RyR), which are modulated by various signals, including luminal and cytoplasmic Ca^2+^, ATP, Mg^2+^, phosphorylation, redox status and electrical signals^106–108^. It is important to note that the opening of ER/SR Ca^2+^ efflux channels is often triggered by increased Ca^2+^ levels at its cytoplasmic surface, originating from a different source (most commonly the influx of extracellular Ca^2+^), a process termed calcium-induced calcium release (CIRC) that serves to amplify the original signal and plays a fundamental role in the function of excitable cells^109^. Moreover, the depletion of calcium from ER/SR can activate the Ca^2+^-sensing stroma interaction molecule (STIM) proteins, located in the ER/SR membrane. Then, activated STIM (STIM1 in vertebrates that possess two homologs) translocates to ER/SR-PM junctions, where it interacts with Orai proteins to form calcium release-activated (CRAC) channels, a highly selective member within the store-operated calcium (SOC) channel family, that allows prolonged influx of extracellular Ca^2+^. This mechanism, collectively referred to as store-operated calcium entry (SOCE), functions to replenish ER/SR stores, while maintaining prolonged high [Ca^2+^]_c_ signals, which cannot otherwise be sustained by internal depots, and has been attributed with essential roles in the physiology of both excitable and non-excitable cells^110^.

Similarly to the ER/SR, the lumen of the GA exhibits high levels of Ca^2+^, which are established by the action of SERCA and secretory pathway Ca^2+^-ATPase (SPCA) pumps^111^. Further highlighting the resemblance between the two compartments, GA-stored calcium can be rapidly released in the cytoplasm through IP_3_R and RyR channels^111^. However, the GA is not homogeneous in terms of Ca^2+^ handling. The *cis*-Golgi is primarily enriched in SERCAs and IP_3_Rs, mobilizing Ca^2+^ in a manner similar to the ER/SR, although with some differences in the kinetics of the response^112,113^. In contrast, Ca^2+^ uptake in the *trans*-Golgi is predominantly mediated by SPCAs, while the ability for its release relies on the presence of RyR channels, which shows significant variations between different cell types^112,113^. Consequently, the *trans*-Golgi appears to be functionally distinct from the ER/SR and can serve either as a reservoir of non-mobilizable Ca^2+^ or as a hub for spatially confined Ca^2+^ release in response to localized CIRC (or other RyR-activating stimuli), depending on the presence or the absence of RyRs.

### Calcium trafficking in endo-/lysosome

Beyond the structurally and functionally related stores of the ER/SR and the GA, significant amounts of Ca^2+^ can also be mobilized from late endosomes/lysosomes. Ca^2+^ concentration in lysosomes ([Ca^2+^]_lys_) is in the 500 μM range, approximately 5000 times higher than those in the cytoplasm and comparable to that of the ER/SR^114^. Such high [Ca^2+^]_lys_, combined with the small cellular volume of the lysosomal compartment, renders lysosomes ideally suited for generating spatially confined Ca^2+^ currents and microdomains for localized responses. Congruently, lysosomal Ca^2+^ efflux has been implicated in various cellular processes, including autophagy and signal transduction, as well as in the biogenesis, acidification and exocytosis of lysosomes themselves^115–120^. The release of Ca^2+^ from lysosomes is mediated mainly by members of two families of cation channels, the transient receptor potential mucolipin channels (TRPMLs) and the two-pore channels (TPCs). TRPMLs are generally non-selective, Ca^2+^ permeable cation channels, belonging to the transient receptor potential (TRP) superfamily. Their function is primarily modulated by phosphoinositide isoforms, with the endosome/lysosome localized 3,5-biphosphate (PI(3,5)P_2_) acting as their agonist and its PM isotype, 4,5-biphosphate (PI(4,5)P_2_), as their antagonist^121^. Nonetheless, reactive oxygen species (ROS) have also been reported to directly activate the family member TRPML1, triggering lysosomal Ca^2+^ release and stimulating Ca^2+^-dependent autophagy and lysosomal biogenesis^122^. On the other hand, TPCs belong to the superfamily of voltage-gated anion channels and were originally identified as nicotinic acid adenine dinucleotide phosphate (NAADP)-activated Ca^2+^ release channels^123^. Later work though classified TPCs as Na^2+^ channels, activated by PI(3,5)P₂, ultimately leading to the conclusion that TPCs alter their selectivity depending on how they are activated^124–127^. Interestingly, the two different modes of TPCs’ activation seem to induce distinct physiological outcomes, with NAADP-mediated activation stimulating an increase of luminal pH, and PI(3,5)P_2_-mediated activation promoting lysosomal exocytosis^126,127^. Additional lysosomal Ca^2+^ channels have also been reported; however, it is not clear whether their presence is universal or restricted to specific cell types^128^. Among those, the purigenic receptor X4 (P2X4), an ATP-gated channel activated by high luminal ATP and alkalization, is perhaps of most interest, as it has been implicated in lysosomal membrane fusion in a calmodulin-dependent manner^129,130^.

### Regulation of mitochondrial calcium levels

Inside mitochondria, the resting calcium concentration ([Ca^2+^]_mit_) is relatively low compared to other internal stores, kept in the 100 μM range, due to the buffering of free Ca^2+^ ions as calcium phosphate^131^. This high buffering capacity in their matrix allows mitochondria to act as Ca^2+^ sinks that can relieve cells from high [Ca^2+^]_c_ when needed. Alterations in [Ca^2+^]_mit_ significantly impact mitochondrial physiology, as key enzymes of the tricarboxylic acid (TCA) cycle, namely pyruvate dehydrogenase, isocitrate dehydrogenase and oxoglutarate dehydrogenase, are Ca^2+^-dependent^132,133^. As a result, elevated [Ca^2+^]_mit_ enhances the production of NADH, FADH2 and ATP, but also leads to increased generation of ROS. The driving force for mitochondrial Ca^2+^ influx is provided by its electrical gradient, as the IMM is highly electronegative, with a potential of approximately −180 mV on the matrix side, compared to the intermembrane space (IMS). The OMM is easily penetrable by Ca^2+^ that enters the IMS via VDAC (also referred to as porin) that is permeable to ions and small molecules (<5 kDa), including phosphate and adenine nucleotides. At membrane voltages close to 0 mV, most VDAC isoforms are typically found in the “open” state of full conductance, which is weakly anion-selective, whereas at higher potentials (beyond ±30–40 mV) they switch to a “closed” state that shows approximately half of the original conductance and favors the transport of cations, including Ca^2+^ ^134,135^. Consequently, the influx of Ca^2+^ in the IMS via VDAC can be affected by the ability of the organelle to maintain its Δψ_m_, through the function of the electron transport chain (ETC), which, in turn, influences the potential across the OMM. Intriguingly, VDAC1 has been found to physically interact with the IP_3_R channel of the ER membrane in mitochondria-ER contact sites (MERCs), through the molecular chaperone glucose-regulated protein 75 (Grp75), an interaction that stimulates VDAC-mediated Ca^2+^ transport and provides a funnel for the direct loading of mitochondria with Ca^2+^ by the ER^136,137^. The efficiency of this Ca^2+^ transport route appears to be highly dependent on the distance between ER and mitochondria; while organelles at MERCs juxtapose at distances that typically range between ~10 and 50 nm, Ca^2+^ transfer seems to require a space of less than 25 nm, exhibiting a maximum peak at ~20 nm^138,139^. Interestingly, the optimal tethering of mitochondria to the ER, which allows efficient Ca^2+^ transport, has recently been shown to be mediated by the interaction of MFNs of the OMM with an ER-localized splice variant of MFN2, termed ERMIT2, revealing a novel aspect of the interconnections between Ca^2+^ trafficking and mitochondrial dynamics^140^. Moreover, it has been reported that the integrity of MERCs involves Selenoprotein N (SEPN1), an ER-localized sensor of luminal Ca^2+^ that can stimulate the activity of SERCA pumps, and thus potentially provide a link between the transport of Ca^2+^ from the ER to mitochondria and the refilling of ER Ca^2+^ stores^141,142^.

Once in the IMS, Ca^2+^ has to be transferred across the IMM, to enter the mitochondrial matrix. Two mechanisms with distinct kinetics have been described for Ca^2+^ influx, although it is not clear whether these are mediated by the same or by different molecular components. The most well-studied mechanism is mediated by a highly selective multiprotein Ca^2+^-conducting channel, the mtCU complex, that requires high [Ca^2+^]_c_ (typically in the low μΜ range) to be activated due to its low affinity (K_D_ = 20–30 μM), and is inhibited by the hexavalent cation ruthenium red and its more specific derivative Ru360^143–145^. The alternative mechanism is termed rapid mode Ca^2+^ uptake (RaM) and functions at [Ca^2+^]_c_ below 200 nM, which is too low to activate the uniporter, but weakens as Ca^2+^ levels rise and can only be reactivated after a short resting period at concentrations below 100–150 nM^146–148^. Due to its characteristics, RaM has been proposed to mediate mitochondrial uptake during low-amplitude Ca^2+^ pulses, allowing the tuning of [Ca^2+^]_mit_ to cytoplasmic Ca^2+^ oscillations. It must be noted though that RaM has only been reported in a limited number of studies, and its biological significance still remains under question.

mtCU is typically composed of the channel-forming mitochondrial calcium uniporter (MCU) protein, the scaffolding protein essential mitochondrial response element (EMRE) and the regulatory mitochondrial calcium uptake (MICU) proteins. Despite the existence of conflicting studies, conducted in different organisms, that report either tetrameric or pentameric stoichiometry, MCU appears to universally assemble into oligomeric transmembrane barrels that span the IMM, with evolutionary conserved amino acid motifs constituting the ion selectivity filter at the IMS apex of the pore^149–152^. Interestingly, high [Ca^2+^]_mit_ disrupts the assembly of monomeric MCU into functional multimeric channels, through its interaction with the protein’s N-terminal matrix domain, suggesting the presence of a self-regulatory mechanism that protects mitochondria from excessive Ca^2+^ loading^153^. Furthermore, most vertebrates possess an MCU paralog, named MCUb, that shares a similar structure and topology with MCU and has the ability to interact with it, forming MCU:MCUb hetero-oligomers^149^. However, MCUb does not exhibit any Ca^2+^-conducting abilities but acts as a dominant-negative inhibitor of mtCU, dramatically reducing MCU activity, probably by disrupting the architecture of the pore^149^. As a result, expression levels of MCUb can serve as a means to regulate mtCU function and therefore the ability of mitochondria to uptake Ca^2+^.

Beyond the pore-forming MCU protein, the function of the mtCU is regulated by the family of MICU proteins, which are characterized by the presence of two highly conserved Ca^2+^ binding EF-hand domains and act as gatekeepers to modulate the permeability of the channel. Among them, MICU1 follows the evolutionary pattern of MCU and physically interacts with it, while the other two members of the family are not as ubiquitously expressed and participate in the complex via their interaction with MICU1^154–157^. MICU1 has been found to play a dual role, ensuring the impermeability of the channel at low [Ca^2+^]_c_, but also facilitating its activation when [Ca^2+^]_c_ rises^158^. Interestingly, it has been proposed that a MICU1-free, ungated form of the uniporter is responsible for RaM under low [Ca^2+^]_c_ conditions, but rising Ca^2+^ levels eventually induce the interaction of MICU1 with MCU and inhibit Ca^2+^ influx until [Ca^2+^]_c_ is high enough to activate the gated form of the channel or low enough to allow for the dissociation of MICU1 from MCU^159^. On the other hand, MICU2 has been shown to form an obligate heterodimer with MICU1 that acts to keep the channel at a closed conformation, while MICU3, which also interacts with MICU1, potentiates the channel for Ca^2+^ uptake, possibly by disrupting MICU1:MICU2 dimers^156,160–162^. Lastly, EMRE is an essential component of metazoan mtCU, that activates MCU through direct binding, while loss of its function or disruption of its interaction with MCU abrogates mtCU-dependent Ca^2+^ uptake in vivo^163–165^. However, EMRE is not present in protozoa, fungi and plants^163^. Its absence from these taxa though does not undermine their uniporter activity compared to animals, suggesting the occurrence of conformational changes in the complex that took place in the evolutionary lineage of metazoa and necessitated the parallel evolution EMRE to maintain mtCU function^164,166^. Besides the mtCU, which is responsible for the main load of Ca^2+^ influx through the IMM, other channels or pumps have also been reported to provide alternative routes. Such proteins may present a means for Ca^2+^ uptake with different kinetics, under conditions that the uniporter is inactive (e.g., RaM), and their existence offers an explanation for the regular resting levels of [Ca^2+^]_mit_ that are often observed in systems with chronic genetic disruption of the mtCU^6^. It has been postulated that mitochondria possess a ryanodine receptor, termed mitochondrial ryanodine receptor (mRyR), with characteristics similar to RyR1, typically found in the SR of skeletal muscle cells^167–169^. Although its existence is debated, the putative mRyR has been suggested to function at [Ca^2+^]_c_ lower than mtCU and mainly mediate microdomain-localized uptake of Ca^2+^ released from other internal stores, using the electrochemical gradient of Ca^2+^ across the IMM to drive its influx^169,170^. In addition, two IMM ion exchangers that normally function to extrude Ca^2+^ from mitochondria have also been reported to function in reverse and mediate Ca^2+^ influx under certain conditions. These are the Na^+^/Ca^2+^ exchanger NCLX and the Ca^2+^/H^+^ antiporter LETM1^171,172^.

NCLX is an electrogenic member of the Na^+^/Ca^2+^ exchanger (NCX) family, as it operates at a 3Na^+^:1Ca^2+^ stoichiometry and stands out as the only one that can also mediate Li^+^/Ca^2+^ exchange^173,174^. Under normal cellular conditions, NCXL functions in forward mode, with the efflux of 1 Ca^2+^ and the influx of 3 Na^+^ molecules resulting in the net gain of 1 positive charge in the matrix of mitochondria. However, under hypoxic conditions, or when mitochondria are depolarized, elevated [Ca^2+^]_c_ can lead to reverse mode function of NCLX, where 1 Ca^2+^ is imported, in exchange for the efflux of 3 Na^2+^ ions^175–177^. Moreover, it has been reported that NCLX can alternate between forward and reverse transport modes in depolarized organelles, generating oscillations of Ca^2+^ levels in the matrix that reflect the fluctuation of [Ca^2+^]_c_^178^. It must be noted though that such observations have been performed under artificial conditions, with depolarization of a magnitude that occurs quite rarely in living cells. As a result, reverse mode mitochondrial Na^+^/Ca^2+^ exchange is unlikely to take place under physiological circumstances, but only under extremely stressful situations, as those induced by ischemia. LETM1 is a H^+^ antiporter that can transport various cations, but its affinity for Ca^2+^ is much higher, suggesting that it is its primary substrate^179^. Its electrogenicity is still debated, as there have been studies supporting both a 1:1 and a 1:2 Ca^2+^/H^+^ stoichiometry^172,179,180^. The study of liposome-reconstituted LETM1 has demonstrated its reversible function, where the direction of transport is predominantly governed by the pH gradient along the two sides of the membrane, and H^+^ moieties move towards the region of higher pH^181^. Additionally, significant differences in the relative concentrations of Ca^2+^ appear to also influence LETM1, shifting its transport direction to balance the ion’s chemical gradient^181^. As a result, the relatively alkaline mitochondrial matrix (pH ≈ 8 compared to pH ≈ 7 in the IMS), renders LETM1 primarily a Ca^2+^ efflux route that can be reversed by a drop of pH in the matrix or its rise in the cytoplasm and the IMS. Interestingly, LETM1 has been reported to mediate Ca^2+^ influx at cytoplasmic concentrations <1 μΜ, suggesting it could play a significant role in the response of mitochondria to slow [Ca^2+^] increases that are not enough to activate the mtCU^181,182^.

Despite the synergy of NCLX and LETM1, mitochondria can get overly filled with Ca^2+^, a condition that has been found sufficient to induce the formation of an unselective, highly conductive multiprotein channel of the IMM, termed mitochondrial permeability transition pore (mPTP)^183–185^. Although the exact molecular identity of the pore-forming constituents is still a field of active research, mPTP is believed to be structured around the adenine nucleotide translocase (ANT) and the F-ATP synthase, while cyclophilin D has been attributed with a modulatory role in the opening of the channel and the possible involvement of VDACs from the OMM is still debated^186^. While long-lasting openings of the mPTP have a well-documented pathological effect, resulting in mitochondrial depolarization, organelle swelling and ultimately cell death under stressful conditions, its transient, low conductance opening (or “flickering”) has been suggested to play physiological roles by serving as a Ca^2+^ release pathway^187,188^. Whether such short-term openings of the mPTP function simply as a safety valve that relieves mitochondria from detrimental Ca^2+^ overload or if they have additional roles in physiology is still under question.

## Molecular links between calcium signaling and mitophagy

Ca^2+^ signaling is pivotal in various cellular processes, including energy production, cell fate decisions and autophagic pathways. Over the past decade, increasing evidence has underscored its crucial implication in mitophagy, functioning at the intersection of mitochondrial quality control and intracellular signaling. While this intricate interplay remains an emerging area of research, it can be broadly divided into two key aspects. First, factors of the mitophagy pathway have been implicated in the regulation of mitochondrial Ca^2+^ levels, likely to support mitochondrial function and prevent Ca^2+^-induced activation of cell death pathways. Inversely, Ca^2+^ fluctuations across distinct cellular compartments have been suggested to act as signaling cues to modulate distinct steps of the mitophagy process. By integrating these complementary roles, Ca^2+^ signaling plays a central role in preserving mitochondrial integrity and ensuring overall cellular and organismal homeostasis.

### Calcium-dependent regulation of mitochondrial dynamics prior to mitophagy

Mitochondrial dynamics, entailing motility, fission and fusion are essential for the maintenance of mitochondrial function and cellular homeostasis. These interconnected processes modulate mitochondrial distribution, morphology and connectivity, thereby allowing mitochondria to adapt rapidly to cellular stress or to differential energetic demands. Proper coordination of these dynamic pathways is critical for the initiation and progression of mitophagy^189,190^. Mitochondrial motility, orchestrated by motor-adaptor proteins ensures the proper distribution of mitochondria to regions of high energy demands, while it facilitates quality control by transporting damaged or superfluous mitochondria towards the autophagic machinery. The process of fission segregates dysfunctional mitochondria, facilitating their mitophagic degradation, while fusion mitigates stress by diluting defective components within healthy mitochondrial networks, as a means to prevent excessive mitochondrial loss. A growing amount of evidence underscores the pivotal role of calcium signaling in regulating mitochondrial dynamics, suggesting that Ca^2+^ fluxes act as a key modulator of these processes, thereby predisposing mitochondria for mitophagic clearance^191^.

**Fig. 2:**
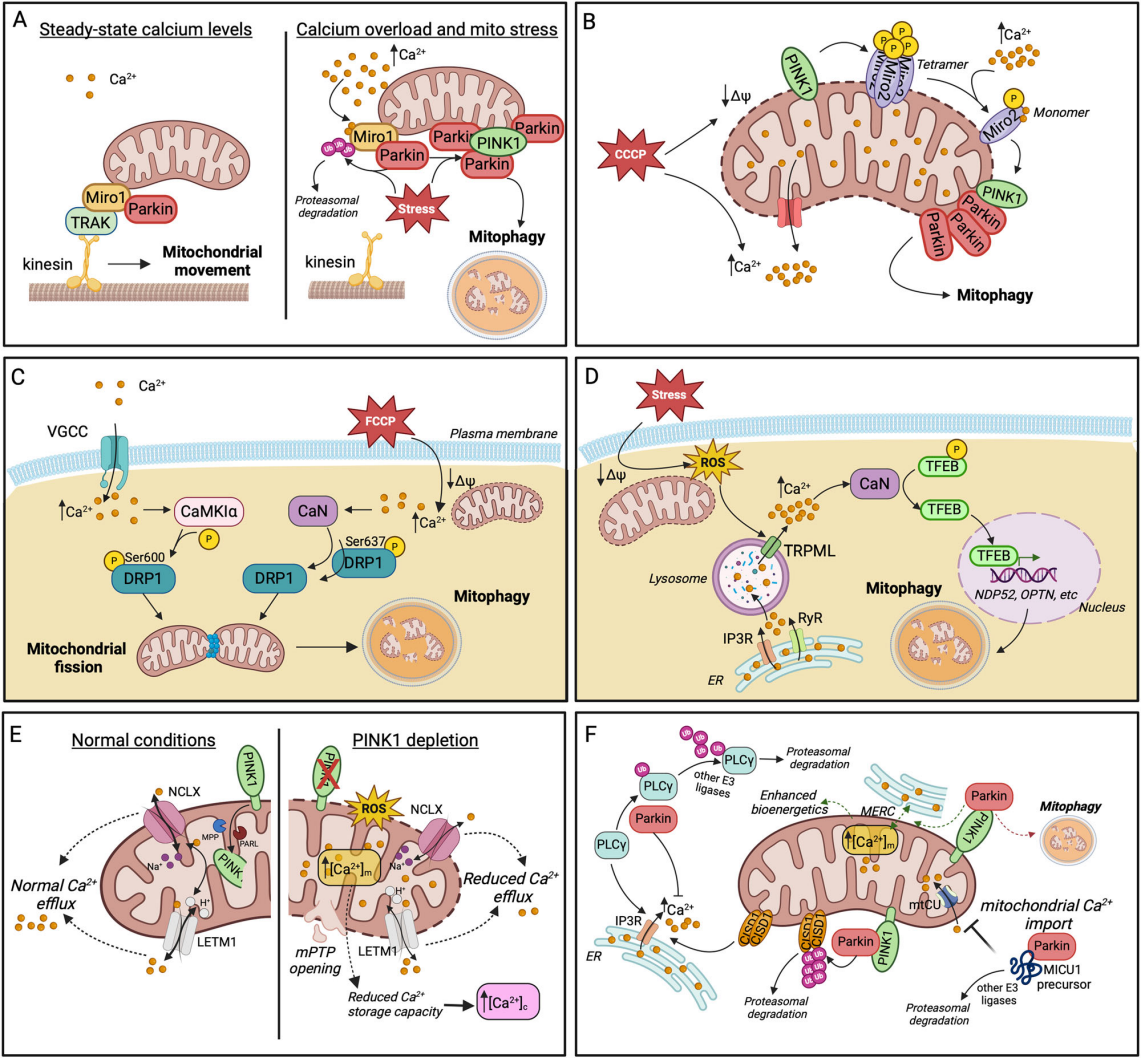
Interplay between calcium homeostasis and mitophagy. **A** At low cytosolic calcium levels, Miro1 facilitates mitochondrial movement by interacting with Milton/TRAK. Increased Ca^2+^ disrupts this interaction, exposing Miro1 to ubiquitination by Parkin and the subsequent PINK1/Parkin-dependent mitophagy upon concurrent mitochondrial stress. **B** CCCP-induced mitochondrial depolarization triggers the demultimerization of Miro2 from a tetramer to a monomer, a process dependent on PINK1 phosphorylation and Ca^2+^ binding to Miro2 EF2 domain. Miro2 re-alignment stimulates Parkin translocation and mitophagy initiation. **C** Cytosolic calcium accumulation activates the kinase CAMKIα and the phosphatase CaN which mediate the phosphorylation (Ser600) and dephosphorylation (Ser637) of DRP1, respectively. Both modifications promote mitochondrial translocation of DRP1 which triggers fission, priming mitochondria for mitophagic degradation. **D** In pancreatic β cells, mitochondrial stress generates ROS that act as signaling molecules for lysosomal calcium release via TRPML channels. Elevated Ca^2+^ activates CaN, which, in turn, dephosphorylates TFEB, enabling its nuclear translocation. In the nucleus, TFEB promotes the expression of the mitophagy adaptor proteins NDP52 and OPTN, among others. Lysosomal calcium stores are primarily replenished from ER. **E** The key mitophagy factor PINK1 facilitates mitochondrial calcium release by phosphorylating NCLX and LETM1 exchangers, thereby preventing Ca^2+^ overload within mitochondria. PINK1 depletion results in mitochondrial calcium overload, increased ROS generation, mPTP opening and a subsequent rise in cytosolic Ca^2+^ due to diminished mitochondrial buffering capacity. **F** Examples of Parkin implication in the regulation of calcium homeostasis. Parkin prevents cytosolic calcium overload by ubiquitinating and promoting the proteasomal degradation of PLCγ, the upstream activator of IP3R. CISD1 has been identified as the downstream effector for Parkin-dependent regulation of ER-calcium release. Parkin is proposed to initially enhance ER-mitochondria tethering and mitochondrial Ca^2+^ influx as a means to boost bioenergetics (dashed green arrows). Failure in restoring bioenergetic imbalance enables Parkin’s mitophagy-inducing activity for the elimination of compromised mitochondria (dashed red arrow). Moreover, the cytosolic fraction of Parkin impedes mitochondrial calcium uptake by interacting with MICU1 precursor, enhancing its ubiquitination and subsequent proteasomal degradation. Created in BioRender. Palikaras, K. (2024) https://BioRender.com/r42n942.

Ca^2+^ plays a critical role in modulating mitochondrial motility and Parkin recruitment prior to mitophagy. Studies in primary neuronal cultures showed that at resting cytoplasmic Ca^2+^ levels, the OMM protein Miro1 interacts with Milton/TRAK adaptor proteins, facilitating mitochondrial movement along the cytoskeleton. In this state, Miro1 forms an initial, non-ubiquitinated complex with a small portion of Parkin at the OMM. When cytosolic calcium levels rise, Ca^2+^ binds to the EF-hand motifs of Miro1, inducing conformational changes that disrupt its interaction with the cytoskeleton, effectively halting mitochondrial movement. Upon concurrent mitochondrial damage, this Ca^2+^ -triggered event leads to the accumulation of PINK1 to the OMM and the activation of Parkin. In turn, Parkin ubiquitinates proteins of the OMM, including Miro1, and stimulates the mitophagic process (Fig. 2Α)^192–194^. Notably, the exact source of the localized Ca^2+^ increase that drives mitochondrial immobilization prior to mitophagy has not been well defined. A possible mechanism could involve enhanced Ca^2+^ efflux from mitochondria, likely due to intrinsic mitochondrial damage. Alternatively, transient Ca^2+^ influx from extracellular sources during synaptic activity or neuronal firing could raise calcium levels locally, particularly at active zones, where Ca^2+^ channels are highly concentrated. This elevation might serve to immobilize mitochondria in order to locally enhance their Ca^2+^ buffering capacity or facilitate their selective elimination through mitophagy. In this context, syntaphilin (SNPH) might contribute to mitochondria immobilization by displacing kinesin-1 (KIF5) from the Miro-TRAK complex, in response to Ca^2+^ fluctuations triggered by neuronal activity^195^. This switch could, in turn, favor the local elimination of organelles at distal parts of axonal processes against their transport back to the soma for degradation, thus speeding up their clearance and minimizing the risk of dispersing harmful molecules (e.g., ROS, mtDNA release) from damaged organelles.

Interestingly, Miro1 dysfunction has recently been associated with impaired Ca^2+^ buffering and mitochondrial dynamics. Loss-of-function mutations in the N-terminal EF-hand and in the GTPase domains of Miro1 significantly disrupt the formation of ER-mitochondria contact sites, impairing the ability of mitochondria to sufficiently buffer excess Ca^2+^ originated from the ER or the cytosol. The consequent reduction in mitochondrial Ca^2+^ levels adversely affects mitochondrial bioenergetics, triggering the activation of mitophagic pathways to eliminate the dysfunctional mitochondria^196^. Another study in mouse embryonic fibroblasts (MEFs) provided evidence for a Ca^2+^-dependent regulatory mechanism involving Miro2/RHOT2 as an early event preceding mitophagy induction. Specifically, in response to CCCP-induced depolarization, the OMM-resident Miro2 undergoes a shift from a tetramer to a monomer. This demultimerization is driven by two critical events: PINK1-mediated phosphorylation of Miro2 at Ser325/Ser430 residues and Ca^2+^ binding in the EF2 domain of Miro2. The resulting monomeric form of Miro2 is re-aligned to the OMM and is suggested to function as a platform for the precise recognition and translocation of Parkin to damaged and Ca^2+^-releasing mitochondria. Similar to the mechanism described for Miro1, Ca^2+^ binding to the EF-hand motifs of Miro2 may also serve as a mechanism for mitochondrial immobilization, facilitating the selective removal of dysfunctional mitochondria through the PINK1/ Parkin pathway (Fig. 2B)^197^.

Mitochondrial fission is also a Ca^2+^-dependent process that segregates mitochondria, facilitating their mitophagic removal. Ca^2+^ influx through VGCCs has been shown to cease mitochondrial motility and induce fission in cultured hippocampal rat neurons. Mechanistically, elevated calcium activates CaMKIα kinase, which, in turn, phosphorylates the fission-related protein DRP1 at Ser600, enhancing its recruitment to mitochondria for the initiation of fission^198^. Additionally, calcineurin (CaN), a Ca^2+^ and calmodulin-dependent serine/threonine protein phosphatase has also been involved in the modulation of DRP1 function. Cytosolic Ca^2+^ elevation in response to mitochondrial depolarization activates CaN, which dephosphorylates DRP1 at Ser637. This dephosphorylation facilitates DRP1 translocation to mitochondria, driving mitochondrial fragmentation (Fig. 2C)^199^. Future studies are needed to delineate whether these calcium-dependent post-translational modifications on DRP1 have a direct or indirect impact on mitophagy per se.

A similar mechanism involving CaN has been reported in mouse and nematode models of Autosomal Dominant Optic Atrophy (ADOA), characterized by loss-of-function mutations in the fusion-related Mitochondrial Dynamin Like GTPase optic atrophy 1 (OPA1). OPA1 depletion results in substantial cytoplasmic Ca^2+^ elevation that enhances mitochondrial fragmentation and mitophagic elimination in a process dependent on the synergistic function of CaN and AMPK^200^. Though, further research is required to clarify the precise role of Ca^2+^-dependent CaN in the modulation of OPA1 function and whether Ca^2+^ has a more direct role in modulating mitochondrial fusion prior to mitophagy.

Bidirectionally, alterations in mitochondrial dynamics influence Ca^2+^ homeostasis in distinct subcellular compartments. A study conducted in mouse skeletal muscle fiber cells demonstrated that disruption of the fission machinery through DRP1 loss impairs the tethering between sarcoplasmic reticulum (SR) and mitochondria. This disruption results in abnormally large mitochondria with a substantially increased capacity for Ca^2+^ uptake. Enhanced Ca^2+^ uptake into mitochondria depletes the cytoplasmic Ca^2+^ pool, essential for muscle contraction, ultimately compromising muscle function and eventually leading to muscle fiber death^201^. The detrimental effects on muscle viability are suggested to be mediated by impaired mitophagy and the opening of mPTP, which triggers the release of pro-death factors^201^. These mechanisms may also be relevant to neuronal physiology. Disruption of mitochondrial fission and ER-mitochondria tethering in neurons could similarly impair calcium buffering, particularly in regions with high Ca^2+^ flux, such as synaptic terminals and dendritic spines. Dysregulation of local calcium dynamics in these compartments could exacerbate neuronal stress and contribute to synaptic dysfunction, a major hallmark of neurodegenerative diseases. Furthermore, impaired mitophagy and mPTP opening in neurons may play a comparable role to that observed in muscle cells, promoting mitochondrial dysfunction and cell death mechanisms.

### The CaN-TFEB pathway for mitophagy induction

An intriguing connection between mitophagy and Ca^2+^ homeostatic pathways has been recently reported in pancreatic β cells. Exposure to mitochondrial stressors triggers the generation of ROS, which, in turn, act as signaling molecules to enhance calcium release from lysosomes. This surge in cytosolic Ca^2+^ levels activates CaN, an upstream positive regulator of transcription factor EB (TFEB). CaN-dependent dephosphorylation of TFEB triggers its translocation to the nucleus, where it drives the expression of target genes, including the mitophagy receptors NDP52 and OPTN, alongside other factors involved in lysosomal biogenesis and autophagic pathways. This sequence of events ultimately leads to the induction of mitophagy for the elimination of mitochondrial stress. In parallel, lysosomal Ca^2+^ stores are replenished by ER-derived Ca^2+^, likely facilitated by ER-lysosome contacts. Moreover, ER Ca^2+^ depletion triggers store-operated Ca^2+^ entry (SOCE) from extracellular sources, ensuring sustained Ca^2+^ homeostasis. This finely tuned mechanism not only supports mitophagy but also preserves mitochondrial function in pancreatic β cells under metabolic stress (Fig. 2D)^202^.

While CaN-dependent modulation of TFEB has been previously involved in the induction of general autophagy^117^, the study in pancreatic cells focuses specifically on mitophagy per se, suggesting a well-defined Ca^2+^-driven mechanism for mitophagy induction. The described CaN-TFEB axis likely corresponds to a much slower procedure for mitochondria elimination, compared to the rapid response of PINK1/Parkin-mediated mitophagy observed upon acute mitochondrial damage. Towards this direction, further investigation is needed to clarify the specific role of Ca^2+^ in the initiation and execution of these distinct mitophagy pathways and to explore whether this Ca^2+^-mitophagy interplay is conserved across different tissues, including the complex neuronal system.

### Interconnection between calcium signaling and factors of the PINK1/Parkin mitophagy pathway

Accumulating evidence supports that specific mitophagy factors are implicated in the modulation of calcium homeostasis. For instance, PINK1 has been shown to regulate mitochondrial Ca^2+^ efflux through the mitochondrial Na^+^/Ca^2+^ exchanger NCLX. PINK1-deficient human neuroblastoma cell lines display mitochondrial Ca^2+^ overload, elevated ROS and rapid opening of the mPTP. Additionally, cytosolic Ca^2+^ content is also substantially increased due to reduced mitochondrial Ca^2+^ buffering capacity (Fig. 2E)^203^. Consistently, purified brain mitochondria from PINK1 knockout mice exhibited elevated mitochondrial Ca^2+^ levels, which subsequently induced membrane permeability transition (mPT)^204^. Additional studies revealed that protein kinase A (PKA) positively regulates NCLX activity through direct phosphorylation at its Ser258 residue^205^. However, the possibility that PINK1 directly interacts with and phosphorylates NCLX remains unresolved. Thus, further studies are required to determine whether PINK1 directly modulates NCLX activity, in addition to its indirect regulation through PKA.

Except for the regulation of Na^+^/Ca^2+^ exchanger, PINK1 is reported to also control Ca^2+^ extrusion from mitochondria through LETM1^206^. LETM1 is an IMM protein, identified to function as a Ca^2+^/H^+^ antiporter, mediating Ca^2+^ influx and efflux in a dose-dependent manner^181,207^. Notably, it is the processed form of PINK1, rather than the full-length protein that phosphorylates LETM1 at Thr192 residue (Fig. 2E). This phosphorylation may act as an early step in mitophagy induction or operate under baseline conditions to ensure proper mitochondrial Ca^2+^ trafficking. This PINK1/LETM1 interaction is suggested to play a neuroprotective role, by alleviating stress-induced damage^206^. In contrast to reports supporting PINK1’s role in preventing mitochondrial Ca^2+^ overload, PINK1-deficient MEFs have been shown to exhibit reduced Ca^2+^ influx into mitochondria, accompanied by moderate mitochondrial depolarization and fragmentation^208^. This finding suggests that the role of PINK1 in the modulation of Ca^2+^ dynamics may be context-dependent, potentially varying across different cell types, tissues or upon distinct stimuli.

Beyond PINK1, Parkin has also been implicated in the modulation of intracellular calcium homeostasis (Fig. 2F). In neuroblastoma SH-SY5Y cell line, as well as in cortical, striatal and nigral regions of post-mortem human brain tissues, Parkin interacts and ubiquitinates phospholipase Cγ1 (PLCγ1), a key upstream regulator of IP_3_R-mediated-Ca^2+^ release from the ER. Τhis ubiquitination enhances PLCγ1 proteasomal degradation, thereby, preventing cytosolic Ca^2+^ overload. Given that Parkin adds only mono-ubiquitin tags in PLCγ1, other E3 ubiquitin ligases may also be involved in this regulatory process^209,210^. A recent study conducted in mammalian cell lines and flies identified the OMM protein CDGSH iron sulfur domain 1 (CISD1) as a downstream effector of Parkin-mediated regulation of ER Ca^2+^ signaling. Specifically, Parkin ubiquitinates CISD1, targeting it for proteasomal degradation and, thereby, disrupting its function as a positive regulator of IP_3_R^211^. This evidence suggests that Parkin-dependent inhibition of ER calcium release could play a role either in facilitating the PINK1/ Parkin mitophagy pathway, once activated, or as part of a feedback mechanism, signaling the termination of mitophagy. Further investigations are needed to elucidate the precise role of ER-derived Ca^2+^ during the different phases of mitophagy (initiation, execution, termination), and to determine whether Ca^2+^ import into mitochondria is essential at each stage. Supporting this notion, another proposed calcium-regulatory mechanism involving Parkin suggests that, under basal conditions, Parkin enhances ER-mitochondria connectivity, leading to increased Ca^2+^ influx into mitochondria. This process serves as a first line of defense by boosting mitochondrial bioenergetics in response to potential dysfunction. If mitochondrial damage persists, Parkin shifts its function towards the activation of mitophagy to eliminate the compromised mitochondria^212^. Another study conducted in MEFs and fly models of mitochondrial dysfunction, highlighted the essential role of the Ca^2+^-dependent phosphatase CaN in Parkin translocation to damaged mitochondria and the subsequent PINK1/Parkin-mediated mitophagy^213^, suggesting that CaN is also implicated in mitophagy initiation, rather than only affecting mitochondrial dynamics in response to cytosolic Ca^2+^elevation.

Additionally, Parkin has been implicated in the regulation of the mtCU complex by controlling basal levels of the MICU1 regulator. Specifically, the cytosolic fraction of Parkin interacts with the pre-import precursor MICU1 via its UBL domain, rather than its E3 ligase domain and facilitates MICU1 ubiquitination and subsequent proteasomal degradation, likely in collaboration with other E3 ubiquitin ligases. By controlling MICU1 levels, Parkin indirectly influences the activity of the entire mtCU complex, including the MICU2 gatekeeper, due to changes in the stoichiometry of MICU1/MICU2 heterodimers^214^. Although the studies mentioned above do not provide clear mechanistic insights into the specific role of Ca^2+^ in mitophagy, they offer valuable clues that could guide future research activities. Notably, these findings underline the importance of further investigating ER-mitochondria connectivity and the dynamics of Ca^2+^ exchange between these organelles, particularly in the context of mitophagy regulation.

## Calcium dysregulation and mitophagy in neurodegeneration

Neurons are highly polarized cells with excessive bioenergetic demands that can vary among different subcellular compartments or in response to stimuli. As a result, the maintenance of healthy mitochondria is of vital importance for their function and their survival. Due to their post-mitotic nature, neurons have to rely heavily on intracellular quality control mechanisms, including mitophagy, in order to preserve a functional organelle pool^215^. In line with such specialized demands, emerging evidence indicates that the regulation of autophagy and mitophagy exhibits some unique characteristics in neuronal cells, compared to other cell types^215–217^. Nevertheless, and despite the existence of compartment-specific variations or alterations in the kinetics of the response, stemming from their complex cellular architecture, the basic principles and molecular components of mitophagy appear to be conserved in neurons^215^.

Interestingly, the autophagic clearance of neuronal mitochondria targets not only damaged organelles but also occurs constitutively. This ongoing process is thought to prevent mitochondrial accumulation and the activation of pro-inflammatory pathways, which are implicated in neurodegeneration^218^. Whether dysregulated neuronal Ca^2+^ dynamics influence this form of constitutive mitophagy or are restricted to the clearance of damaged organelles remains an open question and an area of active research. As a universal second messenger, Ca^2+^ plays critical roles that require tight regulation of its subcellular distribution across various cell types. In neurons, however, Ca^2+^ is intricately involved in cell type-specific physiological processes, such as the transmission of depolarizing signals, neurotransmitter release, and synaptic function. Neuronal excitability relies on the precisely controlled influx of Ca^2+^ through plasma membrane channels, creating a functional dependency that is distinct from other cell types. At the same time, neurons are particularly vulnerable to dysregulated Ca^2+^ fluxes, which are associated with excitotoxicity—a hallmark of neurodegenerative disorders. To accommodate these specialized demands, neurons rely heavily on Ca^2+^-buffering and -sensing proteins, such as calmodulin (CaM), members of the NCS family, calretinin, parvalbumin, and synaptotagmin. Despite these unique adaptations, the channels mediating Ca^2+^ import and its intracellular distribution remain conserved between neurons and other cell types.

**Fig. 3:**
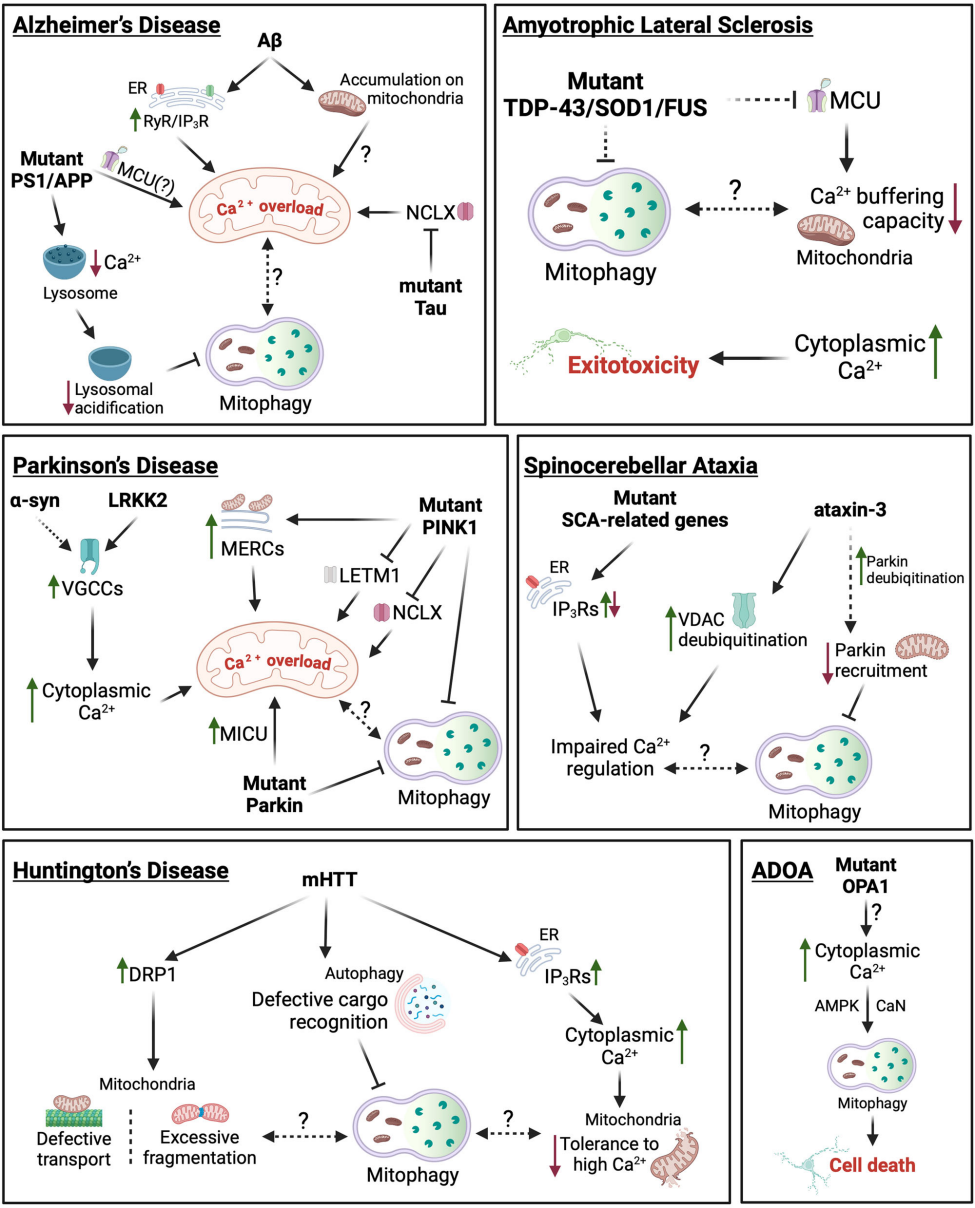
Links between Ca^2+^ homeostasis and mitophagy in the context of neurodegenerative disorders. Dashed lines indicate putative links. Continuous lines correspond to links supported by experimental evidence. For details see “Calcium dysregulation and mitophagy in neurodegeneration.” Created in BioRender. Borbolis, F. (2024) https://BioRender.com/e98r825.

In recent years, deregulated or impaired mitophagy has emerged as a prominent feature in several neurodegenerative conditions^219,220^. Hence, it is not surprising that the intricate relationship between Ca^2+^ regulation and mitophagy in the context of neurodegeneration is attracting increasing attention. Emerging evidence suggests that disruptions in these processes are not isolated events but instead contribute to a vicious cycle that amplifies neuronal damage and accelerates disease progression. While a comprehensive examination of the connections between Ca^2+^ regulation, mitophagy, and neurodegeneration is beyond the scope of this review, these interactions warrant further investigation. Here, we provide a brief overview of cases where the interplay between Ca^2+^ dysregulation and impaired mitophagy contributes to the pathophysiology of both common and rare neurodegenerative disorders (Fig. 3). For further information on the roles of mitophagy and Ca^2+^ homeostasis in neurodegeneration, readers are encouraged to consult recent reviews that thoroughly address these topics^220–223^.

### Alzheimer’s disease

Alzheimer’s disease (AD) is the most common form of dementia, characterized by the accumulation of amyloid-beta (Aβ) plaques and neurofibrillary tangles composed of hyperphosphorylated tau protein^224^. The formation of such protein aggregates is associated with severe mitochondrial dysfunction, which is manifested as impaired energy production, increased oxidative stress and is accompanied by the accumulation of defective mitochondria^225^. Αβ oligomers have been shown to stimulate both Ca^2+^ entry inside neurons and its release from the ER through RyR and IP_3_R channels, leading to mitochondrial Ca^2+^ overload. This triggers the opening of the mPTP, causing mitochondrial depolarization and the release of pro-apoptotic factors, ultimately inducing cell death^226,227^. Moreover, Αβ has been reported to directly accumulate on synaptic mitochondria, inducing the rise of [Ca^2+^]_mit_ and causing the dissipation of their Δψ_m_^228^. Likewise, transgenic mice expressing AD-related mutant forms of amyloid-beta precursor protein (APP) and presenilin (PS1) have been reported to exhibit Aβ-related mitochondrial Ca^2+^ overload, that is ameliorated upon MCU inhibition, while pathological forms of tau have been shown to inhibit NCLX, further sensitizing mitochondria to Ca^2+^ overload^229,230^. The well-documented inability of AD neurons to effectively clear damaged mitochondria due to inefficient Parkin translocation and abnormal acidification of lysosomes further exacerbates their sensitivity to Ca^2+^ spikes^231,232^. Interestingly, mutations in PS1 have been reported to reduce lysosomal Ca^2+^ levels, a condition associated with impaired organelle acidification, indicating a reciprocal relationship between impaired mitophagy and dysregulated Ca^2+^ dynamics that establishes a highly dysfunctional environment, ultimately leading to neurodegeneration^233^. Therefore, therapeutic strategies aimed at restoring calcium homeostasis or enhancing mitophagy are of great promise in mitigating mitochondrial dysfunction and slowing disease progression in AD. Given the limited success of AD treatments focused on targeting Aβ plaques or Tau tangles, modulating mitochondrial Ca^2+^ levels by targeting influx and efflux pathways (e.g., MCU, NCLX, or LETM1) presents a promising alternative approach that warrants further investigation.

### Parkinson’s disease

Parkinson’s disease (PD) is the second most common neurodegenerative disorder, characterized by the loss of dopaminergic neurons in the substantia nigra and the presence of Lewy bodies, intracellular aggregates primarily composed of α-synuclein^234^. Dopaminergic neurons are particularly vulnerable to calcium dysregulation due to their reliance on VGCCs for maintaining their pacemaking activity^235^. Excessive influx through these channels has the potential to induce mitochondrial Ca^2+^ overload, triggering mPTP opening, and causing mitochondrial depolarization. Intriguingly, evidence suggests that mutated α-synuclein can deregulate the function of Ca^2+^ channels and lead to increased cellular Ca^2+^ levels, excessive Ca^2+^ uptake by mitochondria and the collapse of their Δψ_m_^236–238^. Similarly, the activity of VGCCs can also be regulated by leucine-rich repeat kinase 2 (LRKK2), commonly associated with inherited forms of PD^239^. In this setting, impairments in mitophagy, which constitute a main aspect of PD pathogenesis, would deprive cells of a means to remove depolarized organelles, thus potentiating neuronal death.

Supporting this notion, PD-causing mutations in PINK1 have been associated with mitochondrial Ca^2+^ overload in dopaminergic neurons^205,238^. Additionally, PINK1 has been reported to phosphorylate LETM1, promoting its function in Ca^2+^ efflux, and has also been suggested to facilitate NCLX activation, presumably through phosphorylation by PKA^203,205,206^. As a result, PINK1-deficient cells appear to have a lower threshold for the opening of mPTP. Furthermore, mutant PINK1 has been reported to enhance MERCs through the action of Miro and induce the elevation of [Ca^2+^]_mit_, eventually resulting in mitochondrial enlargement and neuronal death^240^. A recent study using iPSC-derived dopaminergic PD neurons has described a localized increase of Ca^2+^ levels in close proximity to the OMM that is sensed by Miro1 and is abrogated when MCU activity is inhibited by Ru360^241^. Combined with previous evidence showing that mitochondria in PD neurons accumulate abnormally high levels of Miro1, delaying mitophagy and that reducing Miro1 levels can mitigate neurodegeneration in PD models, these findings highlight proteins involved in mitochondrial Ca^2+^ flow as promising targets for anti-PD therapies^242,243^.

Interestingly, genetic disruption or pharmacological inhibition of MCU can rescue the death of dopaminergic neurons in *pink1* mutants of both zebrafish and *Drosophila*^244,245^. On the other hand, Parkin has been reported to promote the degradation of MICU1, suggesting the existence of more complex interactions between mitophagy components and mitochondrial Ca^2+^ regulation in PD that have to be taken into account when considering the development of novel therapeutic interventions based on Ca^2+^ dynamics^214^.

### Amyotrophic lateral sclerosis

Amyotrophic lateral sclerosis (ALS) is a fatal neurodegenerative disease characterized by the progressive degeneration of motor neurons that results in muscle weakness, paralysis, and eventually respiratory failure^246^. Most proteins that have been linked to familial or sporadic ALS [e.g., TAR DNA-Binding Protein 43 (TDP-43), superoxide dismutase 1 (SOD1), Fused In Sarcoma (FUS), Chromosome 9 Open Reading Frame 72 (C9orf72)] have been found to interact with mitochondria, while the presence of dysfunctional organelles and impaired mitophagy are considered as hallmarks of the disease^247^. Evidence from various experimental systems suggests that mitochondria from ALS neurons have limited Ca^2+^ buffering capacity, due to inefficient Ca^2+^ uptake^248–251^. As a result, [Ca^2+^]_c_ remains abnormally high after glutamate stimulation, leading to excitotoxicity, a well-documented pathological aspect of ALS that results in neuronal loss^251,252^. Interestingly, MCU is downregulated in motor neurons of asymptomatic or early-disease stage mice expressing ALS-linked SOD1 but is upregulated in the surviving neurons of late-stage animals, suggesting that those neurons that maintain the highest mitochondrial Ca^2+^ buffering capacity are the most resilient to cell death^253,254^. In this setting, it is tempting to speculate that impaired mitophagy plays a causal role in the inability of ALS neurons to buffer Ca^2+^ effectively by hindering the maintenance of a high-quality organelle pool, capable of handling elevated levels of Ca^2+^ uptake. Such a direct connection between mitophagy, Ca^2+^ handling and excitotoxicity in ALS though is still lacking.

### Huntington’s disease

Huntington’s disease (HD) is an autosomal dominant neurodegenerative disorder caused by a CAG repeat expansion in the huntingtin HTT gene, which encodes the huntingtin protein. The mutant huntingtin protein (mHTT) holds a polyglutamine (polyQ) tract in its amino-terminal region, is unable to fold properly and is highly prone to aggregation^255^. Clinically, HD is characterized by motor dysfunction, cognitive decline and psychiatric disturbances caused by the atrophy of GABAergic medium spiny neurons in the striatum and other regions of the brain^256^. mHTT has been reported to directly interact with IP_3_Rs and facilitate their activation, thus potentiating Ca^2+^ release from the ER^257^. Additionally, mitochondria from HD-affected neurons have been shown to exhibit reduced tolerance to high Ca^2+^ loads, which stimulate mPTP opening, Δψ_m_ dissipation and mitochondrial Ca^2+^ release much more readily than in organelles from wild-type cells^258–260^. Interestingly, mHTT has also been found to interact with DRP1 and enhance its activity, causing excessive mitochondrial fragmentation and defective axonal transport in HD neurons^261^. On the other hand, mHTT has been shown to interfere with the cargo recognition step of selective autophagy, including mitophagy, leading to the accumulation of damaged mitochondria, and possibly the inability to clear such fragmented organelles, which, in turn, can increase oxidative stress and contribute to the neurodegeneration observed in the disease^262,263^. Whether these deficiencies in mitochondrial clearance and transport are related to the reduced Ca^2+^ buffering capacity of HD neurons is still under question.

### Spinocerebellar ataxia

Spinocerebellar ataxia (SCA) is a group of genetically heterogeneous neurodegenerative disorders characterized by progressive ataxia, which manifests as a loss of coordination, balance, and motor control. SCAs are caused by mutations in various genes, with each subtype (e.g., SCA1, SCA2, SCA3, etc.) associated with different genetic mutations. These mutations often result in the production of toxic proteins that accumulate within neurons, particularly in the cerebellum and brainstem, leading to neuronal dysfunction and degeneration^264^. Although SCA-related genes exhibit diverse functions, accumulating evidence supports the existence of common pathophysiological mechanisms. Among these, altered Ca^2+^ homeostasis in Purkinje cells (PCs) of the cerebellum has been proposed as a possible pathological SCA trigger^265^. Genetic mutations in IP_3_Rs have been identified as the cause of SCA15/16 and SCA29, while altered function of these channels has been reported in other types of the disease, including SCA1, SCA2 and SCA3^266–269^. While mitophagy in SCA has not gained the same focus as in other neurodegenerative disorders, a recent transcriptomic analysis identified mitochondrial damage and ineffective clearance of organelles as important contributing factors of SCA6 progression^270^. Moreover, ataxin-3 (mutated in SCA3) has been shown to deubiquitinate Parkin, while fibroblasts from SCA3 patients exhibit impaired recruitment of Parkin to depolarized mitochondria, suggesting the disruption of mitophagy^271,272^. Interestingly, VDAC1 was recently identified as an additional deubiquitination target of ataxin-3^272^. However, direct links between deregulated Ca^2+^ and mitochondrial dysfunction in the context of SCA have yet to be established. Further research is needed to assess the impact of SCA-related Ca^2+^ dysregulation on mitophagy and its potential contribution to disease pathogenesis.

### Autosomal dominant optic atrophy

Autosomal dominant optic atrophy (ADOA) is a rare neurodegenerative disease, clinically characterized by the bilateral loss of vision in early childhood or adolescence, due to the degeneration of the RGCs that form the optic nerve^273^. In some cases however, ADOA manifests in a syndromic form that involves multisystemic pathologies, including deafness, ataxia and myopathies^273^. The primary cause of the disease has been linked to mutations in OPA1, a mitochondrial protein that orchestrates IMM fusion and cristae organization, while also participating in the regulation of mitochondrial Ca^2+^ dynamics^274–276^. Studies in the nematode *C. elegans* and in primary cultures of RGCs from murine ADOA models demonstrated that mutant OPA1 is associated with severe depletion of mitochondria from axonal processes, postulated to occur as the result of excessive mitophagy^200,277^. In parallel, ADOA neurons have been found to exhibit elevated Ca^2+^ levels, that activate AMPK and CaN signaling, ultimately inducing mitophagy^200^. Although the source of excessive Ca^2+^ is still undetermined, its chelation has been shown to rescue axonal mitochondrial content of ADOA RGCs and ameliorate various pathological aspects of ADOA-modeling nematodes, thus unraveling a direct link between Ca^2+^ regulation and mitophagy in the pathogenesis of the disease^200^. Further research into these connections may uncover calcium channels and transporters with dysregulated function in the context of the disease, offering promising targets for pharmacological interventions aimed at alleviating symptoms.

## Conclusions and perspectives

The intricate relationship between Ca^2+^ regulation and mitophagy is emerging as a pivotal factor in the pathogenesis of numerous neurodegenerative diseases. Disruptions in these tightly linked processes contribute to mitochondrial dysfunction, heightened oxidative stress, and progressive neuronal loss, underscoring their significance in disease progression. As research in this area advances, the growing body of evidence suggests that restoring calcium homeostasis and modulating mitophagy hold great therapeutic potential.

Although monitoring fluctuations of such a versatile and abundant ion as Ca^2+^ remains a significant challenge, current literature suggests that cytosolic and mitochondrial Ca^2+^ play distinct but interconnected roles in the mitophagy pathway. Localized increases in cytosolic Ca^2+^, particularly within microdomains between subcellular compartments, may regulate mitochondrial dynamics and motility, thereby predisposing mitochondria for mitophagy. These localized Ca^2+^ signals may also modulate key mitophagy components, facilitating subsequent steps in the process. On the other hand, increased Ca^2+^ influx into mitochondria and mitochondrial Ca^2+^ overload, often induced by damage, may serve as an initiating signal for mitophagy pathways. This suggests that local cytosolic Ca^2+^ fluctuations could act as a mechanism to regulate mitophagy in a manner aligned with broader cellular demands and conditions, while mitochondrial Ca^2+^ concentrations may function more specifically as indicators linking mitophagy to the status of individual organelles. This dual role underscores the complexity of the interplay between Ca^2+^ signaling and mitochondrial quality control mechanisms, emphasizing the need for further investigation to unravel these relationships. Future research focused on elucidating the precise molecular mechanisms underlying this interplay will be essential for developing innovative therapeutic strategies. Targeting critical pathways involved in mitochondrial turnover and calcium handling holds promise for treatments that not only alleviate symptoms but also address the root causes of neurodegeneration. Furthermore, manipulating specific components of mitochondrial Ca^2+^ transport systems and mitophagic pathways presents exciting opportunities for interventions aimed at slowing, halting, or potentially reversing disease progression.

The field is moving toward a more holistic understanding of how mitochondrial health and Ca^2+^ signaling interact within the context of neurodegeneration. By integrating insights from molecular biology, pharmacology, and clinical research, future therapeutic strategies may offer a transformative approach, shifting the focus from symptom management to the prevention of neuronal damage and the preservation of cognitive and motor functions. As research progresses, this dynamic area promises to redefine the landscape of neurodegenerative disease treatment, offering hope for improved patient outcomes and a better quality of life.

## Data Availability

No datasets were generated or analyzed during the current study.
